# Patient and Provider Perspectives on Pediatric Telemedicine During the COVID-19 Pandemic

**DOI:** 10.1089/tmr.2021.0032

**Published:** 2021-12-28

**Authors:** Sophie E. Katz, Preston Spencer, Christine Stroebel, Lora Harnack, Jason Kastner, Ritu Banerjee

**Affiliations:** ^1^ Department of Pediatrics, Division of Pediatric Infectious Diseases, Vanderbilt University Medical Center, Nashville, Tennessee, USA.; ^2^ Cumberland Pediatric Foundation, Nashville, Tennessee, USA.; ^3^ VIP Midsouth Children's Clinics, Nashville, Tennessee, USA.

**Keywords:** COVID-19, general pediatrics, satisfaction, telemedicine

## Abstract

The COVID-19 pandemic led to rapid expansion of telemedicine services. We surveyed parent/guardians from March 10 to June 29, 2020, in an academic and community pediatric practice, and community pediatric providers from June 5 to July 13, 2020, to better understand their perceptions of telemedicine and compare parent/guardian satisfaction between in-person and telemedicine encounters. Overall patient satisfaction scores were high in both settings and did not differ between in-person and telemedicine visits (community setting: 93.36 ± 12.87 in-person vs. 88.04 ± 22.04 telemedicine; academic setting: 92.25 ± 11.2 vs. 95.37 ± 8.21). Most providers (82.5%) would be willing to use telemedicine in a nonpandemic situation. Telemedicine should remain available for primary care pediatrics during and after resolution of the pandemic.

## Introduction

The COVID-19 pandemic led to rapid expansion of telemedicine services. We sought to understand parent/guardian (hereafter referred to as parents) and provider perceptions of telemedicine and compare parent satisfaction between in-person and telemedicine encounters. We hypothesized that parents and providers would be satisfied with telemedicine.

## Methods

We used electronic surveys (distributed using REDCap, Vanderbilt University) to assess parent satisfaction at community and academic pediatric practices in middle Tennessee for encounters between March 10 and June 29, 2020. At one community pediatric practice with eight sites, we used a modified version of the Hospital Consumer Assessment of Healthcare Providers and Systems Survey.^1^ We modified the survey language for use in the outpatient setting and only included questions relevant to outpatient visits.

At three pediatric primary care clinics and one adolescent clinic at Vanderbilt Children's Hospital, a tertiary care teaching hospital, we collected data from Press-Ganey^®^ surveys,^2^ which are automatically distributed to all patients seen at this academic medical center. These clinics at Vanderbilt Children's Hospital are referred to as the “academic setting.” While all respondents provided a visit satisfaction score, they were not forced to complete all questions of the Press-Ganey survey.

Electronic provider surveys were sent to community providers from June 5 to July 13, 2020. All respondents had patient visits both in-person and using telemedicine. Provider surveys were not done at the academic practice.

Chi-squared and Student's *t*-tests were used to compare responses by visit type. The study was approved by the Vanderbilt University Institutional Review Board, and a waiver of consent for study participation in surveys was granted.

## Results

### Parent surveys

In the community practice, 2800 surveys were sent to parents during the study period (1475 [52.68%] in-person and 1325 [47.32%] telemedicine); 150 (5.36%) parents responded. The distribution of visit types among survey respondents was 127 (84.67%) in-person visits and 23 (15.33%) telemedicine visits. Compared with children with telemedicine encounters, more children with in-person visits were younger than 4 years (4 [17.39%] vs. 79 [62.2%], *p* < 0.001) and more had parents younger than 25 years (2 [8.7%] vs. 24 [18.9%], *p* = 0.001; Table 1).

**Table 1.** Results of Parent Satisfaction Surveys

	In-person visits, n (%)	Telemedicine visits, n (%)	p
Community pediatrics survey (in-person *n* = 127, telemedicine *n* = 23)			
Demographics			
Female sex—patient	63 (49.61)	13 (56.52)	0.54
Female sex—guardian	121 (95.28)	23 (82.14)	0.29
Patient age (years)			<0.001
<1	37 (29.13)	0	
1–4	42 (33.07)	4 (17.39)	
5–10	32 (25.2)	9 (39.13)	
11–14	11 (8.67)	7 (30.43)	
>15	5 (3.94)	3 (13.04)	
Guardian age (years)			0.001
<18	11 (8.66)	1 (4.35)	
18–24	13 (10.24)	1 (4.35)	
25–34	70 (55.12)	6 (26.09)	
35–44	27 (21.26)	8 (34.78)	
>45	5 (3.94)	7 (30.43)	
Prefer not to answer	1 (0.79)	0	
Guardian level of education			0.69
Did not graduate high school	5 (3.94)	1 (4.35)	
High school graduate	27 (21.26)	4 (17.39)	
Some college or 2-year degree	57 (44.88)	8 (34.78)	
4-year college graduate	19 (14.96)	6 (26.09)	
More than 4-year college graduate	14 (11.02)	4 (17.39)	
Prefer not to answer	5 (3.94)	0	
Non-Hispanic or Latino	119 (93.7)	18 (78.3)	0.13
White	114 (89.76)	20 (86.96)	0.69
Black	17 (13.39)	3 (16.67)	0.97
Survey questions			
The provider gave easy to understand information about health concerns			0.74
Yes, definitely	105 (82.67)	18 (78.26)	
Yes, somewhat	4 (3.15)	1 (4.35)	
No response	18 (14.17)	4 (17.39)	
The provider listened carefully to me			0.43
Strongly agree	107 (84.25)	17 (73.91)	
Agree	17 (13.39)	4 (17.39)	
Disagree	2 (1.57)	0	
No opinion	1 (0.79)	1 (4.35)	
The provider spent enough time with my child			0.91
Strongly agree	103 (81.1)	5 (21.74)	
Agree	20 (15.75)	3 (13.04)	
Disagree	1 (0.79)	0	
No opinion	3 (2.36)	1 (4.35)	
Visit satisfaction score (mean ± SD)	93.36 ± 12.87	88.04 ± 22.04	0.12
Satisfaction score if antibiotic prescribed	93.42 ± 21.58 (*n* = 12)	94 ± 2.83 (*n* = 2)	0.97
Satisfaction score if antibiotic not prescribed	93.35 ± 11.73 (*n* = 111)	87.45 ± 23.08 (*n* = 20)	0.08
Academic pediatrics survey (in-person *n* = 2800, telemedicine *n* = 822)			
Care provider explanation of problem or condition (in-person *n* = 188, telemedicine *n* = 80)			0.66
Very good	159 (84.57)	73 (91.25)	
Good	25 (13.3)	6 (7.5)	
Fair	3 (1.6)	1 (1.25)	
Very poor	1 (0.53)	0	
Care provider efforts to include in decisions (in-person *n* = 188, telemedicine *n* = 79)			0.46
Very good	159 (84.57)	72 (91.14)	
Good	22 (11.7)	7 (8.86)	
Fair	3 (1.6)	0	
Poor	3 (1.6)	0	
Very poor	1 (0.53)	0	
Care provider concerns for questions/worries (in-person *n* = 188, telemedicine *n* = 81)			0.50
Very good	161 (85.64)	73 (90.12)	
Good	20 (10.64)	7 (8.64)	
Fair	6 (3.19)	1 (1.23)	
Very poor	1 (0.53)	0	
Visit satisfaction score (mean ± SD)	92.25 ± 11.2	95.37 ± 8.21	

SD, standard deviation.

Overall visit satisfaction scores were high and did not differ by encounter type (93.36 ± 12.87 in-person vs. 88.04 ± 22.04 telemedicine, *p* = 0.12) or whether an antibiotic was prescribed (in-person visits with antibiotic 93.42 ± 21.58 vs. without antibiotic 93.35 ± 11.73, *p* = 0.99; telemedicine with antibiotic 94 ± 2.83 vs. 87.5 ± 23.08, *p* = 0.7; Table 1). The majority (22, 95.65%) of the 23 telemedicine respondents strongly agreed or agreed that a telemedicine visit was as effective as an in-person visit, and 19 (82.61%) would utilize telemedicine in the future.

In the academic setting, 14,610 visits (12,796 in-person and 1814 telemedicine) occurred during the study period and 3622 patient surveys were completed (24.79% response rate). Satisfaction scores were high and were similar for in-person (92.25 ± 11.2) and telemedicine visits (95.37 ± 8.21; Table 1). Data collected in the academic setting did not allow linkage of antibiotic prescribing with patient satisfaction scores.

### Provider surveys

The online survey was completed by 40/75 (53%) community providers. Almost all providers (39, 97.5%) felt that telemedicine increased patient access to care (Table 2). Most (28, 70%) felt that they were less likely to prescribe antibiotics in a telemedicine encounter than in an in-person encounter (Table 2). The top concern with using telemedicine was the fear of incomplete patient assessment (Table 2). Most (33, 82.5%) would be willing to use telemedicine in nonpandemic situations.

**Table 2.** Results of Provider Satisfaction Surveys Among Community Pediatricians

	Count of respondents (% of 40), n (%)
Demographics	
Female sex	27 (67.5)
Clinical role	
Physician	37 (92.5)
Nurse practitioner	3 (7.5)
Years in practice (years)	
0–4	8 (20)
5–9	6 (15)
10–19	10 (25)
20–29	14 (35)
30+	2 (5)
Telemedicine questions	
How has telemedicine impacted patient access to care?	
Increased access	39 (97.5)
Decreased access	0
No effect	1 (2.5)
Level of satisfaction—patient communication	
Very satisfied	4 (10)
Satisfied	25 (62.5)
Neither satisfied nor dissatisfied	6 (15)
Somewhat dissatisfied	5 (12.5)
Very dissatisfied	0
Level of satisfaction—external distractions in the PATIENT's environment	
Very satisfied	2 (5)
Satisfied	17 (42.5)
Neither satisfied nor dissatisfied	8 (20)
Somewhat dissatisfied	13 (32.5)
Very dissatisfied	0
Level of satisfaction—external distractions in YOUR environment	
Very satisfied	19 (48.7)
Satisfied	10 (25.6)
Neither satisfied nor dissatisfied	6 (15.4)
Somewhat dissatisfied	3 (7.7)
Very dissatisfied	1 (2.6)
Unknown	1 (2.6)
Impact of telemedicine on likelihood to prescribe antibiotics	
Less likely to prescribe	28 (70)
More likely to prescribe	3 (7.5)
No effect	9 (22.5)
Amount of time spent in visit compared with in-person encounters (including chart review and documentation)	
More time	6 (15.4)
Less time	13 (33.3)
Same amount of time	20 (51.3)
Top concern about using telemedicine	
Incomplete patient assessment	29 (72.5)
Connectivity/technology issues	13 (33)
Overprescribing/parental expectations about antibiotics	4 (10)
Ensuring insurance coverage/reimbursement	4 (10)
Top benefit from using telemedicine	
Accessibility/patient convenience	27 (67.5)
Decreases unnecessary patient exposure to viral disease (i.e., COVID-19)	7 (18)
Children are more relaxed at home	5 (13)

## Discussion

In this evaluation of community and academic pediatric primary care practices during the COVID-19 pandemic, parent satisfaction scores were high and similar for telemedicine and in-person encounters. Community providers were also very satisfied with telemedicine and felt it increased patient access to care.

Parent satisfaction scores did not differ between visits with or without an antibiotic prescription. This finding differs from prior studies showing that higher antibiotic prescribing rates are associated with better patient satisfaction scores.^3–5^ In our study, providers felt they would not prescribe more antibiotics during telemedicine versus in-person visits, in contrast to prior studies.^3–5^ This difference may be because our study involved primary care providers, while other studies involved telemedicine providers who lacked preexisting relationships with patients. Of note, only two telemedicine visits in our study had an antibiotic prescribed.

Patients seen in-person were younger and had younger parents than those with telemedicine encounters. This was likely because providers were more vigilant about the need to assess younger children in-person and perform thorough physical examinations. We do not anticipate that age of parent or child would be a confounder to satisfaction scores. In addition, insurance coverage should not bias our results as most insurance companies covered telemedicine and in-person visits equally during the COVID-19 pandemic.

Our study has limitations. Our response rates were low and may have led to sampling bias. However, we surveyed patients from both community and academic practices, which may increase generalizability. We used two different surveys in the two study settings, which limits direct comparison of responses. We do not have data regarding reason for patient presentation, which may be a confounder for preference of visit type.

Many more of the patient survey respondents were seen in in-patient visits than were seen in telemedicine visits, which may introduce bias. We were not able to link provider perceptions of prescribing behavior with actual antibiotic prescriptions. In the academic setting, we did not assess clinician satisfaction or link patient satisfaction with antibiotic prescribing, which are areas for future research.

Despite these limitations, we observed that patients from community and academic pediatric settings and community-based pediatricians have had positive experiences with telemedicine during the COVID-19 pandemic. Telemedicine should remain available for primary care pediatrics during and after resolution of the pandemic.

## Authorship Contribution Statement

S.E.K. conceptualized and designed the study, analyzed the data, drafted the initial article, and reviewed and revised the article. P.S. conceptualized and designed the study, designed the data collection instruments, coordinated and supervised data collection, and reviewed and revised the article. S.E.K. and L.H. conceptualized and designed the study and reviewed and revised the article. J.K. supervised data collection and reviewed and revised the article. R.B. conceptualized and designed the study and critically reviewed the article for important intellectual content. All authors approved the final article as submitted and agree to be accountable for all aspects of the work.

## Abbreviation Used

SD: standard deviation

## References

[1] HCAHPS: Patients' Perspectives of Care Survey | CMS. Available at https://www.cms.gov/Medicare/Quality-Initiatives-Patient-Assessment-Instruments/HospitalQualityInits/HospitalHCAHPS Accessed July 29, 2020.

[2] Press Ganey Associates.Available at https://www.pressganey.com/ Accessed September 14, 2020.

[3] Foster CB, Martinez KA, Sabella C, et al. Patient satisfaction and antibiotic prescribing for respiratory infections by telemedicine. Pediatrics 2019;144:e20190844. 31371464 10.1542/peds.2019-0844

[4] Martinez KA, Rood M, Jhangiani N, et al. Association between antibiotic prescribing for respiratory tract infections and patient satisfaction in direct-to-consumer telemedicine. JAMA Intern Med 2018;178:1558–1560. 30285050 10.1001/jamainternmed.2018.4318 PMC6584324

[5] Ray KN, Shi Z, Gidengil CA, et al. Antibiotic prescribing during pediatric direct-to-consumer telemedicine visits. Pediatrics 2019;143:e20182491. 30962253 10.1542/peds.2018-2491 PMC6565339

